# Opioid-free anesthesia compared to opioid anesthesia for laparoscopic radical colectomy with pain threshold index monitoring: a randomized controlled study

**DOI:** 10.1186/s12871-022-01747-w

**Published:** 2022-07-29

**Authors:** Guangquan An, Guiying Wang, Bingsha Zhao, Xiaoying Zhang, Zhihan Li, Jianfeng Fu, Xuelian Zhao

**Affiliations:** 1 Department of Second Surgery, the Fourth Hospital of Hebei Medical University, Shijiazhuang, Hebei People’s Republic of China; 2 Present Address: Department of Anesthesia, the Third Hospital of Hebei Medical University, Shijiazhuang, Hebei People’s Republic of China; 3 Department of Anesthesia, the Fourth Hospital of Hebei Medical University, 12 Jiankang Road, Shijiazhuang, 050011 People’s Republic of China; 4 Present Address: Department of Anesthesia, Tianjin Chest Hospital, Tianjin, People’s Republic of China

**Keywords:** Analgesia, Anesthesia, Dexmedetomidine, Analgesics, Opioid, Pain Management, Colorectal surgery, Laparoscopy, Colorectal cancer

## Abstract

**Background:**

Few studies have investigated the depth of intraoperative analgesia with non-opioid anesthesia. This study evaluated whether opioid-free anesthesia can provide an effective analgesia-antinociception balance monitored by the / pain threshold index in laparoscopic radical colectomy.

**Methods:**

We enrolled 102 patients undergoing laparoscopic radical colectomy with general anesthesia. Participants were randomly allocated into two groups to receive opioid-free anesthesia (group OFA) with dexmedetomidine (loading dose with 0.6 μg·kg^−1^ for 10 min and then 0.5 μg·kg^−1^·h^−1^ continuous infusion) and sevoflurane plus bilateral paravertebral blockade (0.2 μg·kg^−1^ dexmedetomidine and 0.5% ropivacaine 15 ml per side) or opioid-based anesthesia (group OA) with remifentanil, sevoflurane, and bilateral paravertebral blockade (0.5% ropivacaine 15 ml per side). The primary outcome variable was pain intensity during the operation, as assessed by the pain threshold index with the multifunction combination monitor HXD- I. Results were analyzed using repeated measures analysis of variance and Student’s *t*-test. The secondary outcomes were wavelet index, lactic levels, and blood glucose concentration during the operation. The visual analog scale (VAS), rescue analgesic consumption, and side-effects of opioids after surgery were further assessed.

**Results:**

One hundred and one patients were included in the analysis. Analysis revealed that the intraoperative pain threshold index readings were not significantly different between the groups from incision to the end of the operation (*P* = 0.06). Furthermore, similar changes in the brain wavelet index readings were observed in the OFA and OA groups. There was no statistical difference in VAS scores between the groups (*P* > 0.05); however, non-opioid anesthesia did reduce the rescue analgesic consumption after operation (*P* < 0.05). In the OFA group, the blood glucose levels increased by 20% compared to baseline and were significantly higher than those in the OA group (*P* < 0.001). The incidences of postoperative nausea and vomiting, urine retention, intestinal paralysis and pruritus were not significantly different from those in the OA group (*P* > 0.05).

**Conclusions:**

This study suggests that compared to the opioid anesthesia regimen, our opioid-free anesthesia regimen achieved an equally effective intraoperative pain threshold index in laparoscopic radical colectomy. The incidence of opioid-related adverse reactions was not different between regimens, and intraoperative blood glucose levels were higher with opioid-free anesthesia.

**Trial registration:**

ChiCTR1900021223, 02/02/2019, Title: " Opioid-free anesthesia in laparoscopic surgery: a randomized controlled trial ". Website: hppts://www.chictr.ogr.cn

## Background

Several clinical studies have challenged the common practice of administering opioids during anesthesia, suggesting that opioid-free anesthesia (OFA) may effectively provide adequate antinociception while hopefully reducing opioid-related side effects [1–5]. However, these studies focused more on the effect of OFA on postoperative analgesia scores (visual analog scale, VAS), rescue analgesic consumption, and incidence of adverse reactions related to opioid analgesics. Few studies have assessed the intraoperative depth of analgesia between non-opioid and opioid anesthesia. Under general anesthesia (GA), nociceptive signals are continuously generated and exert negative physiological consequences on unconscious patients. Inadequate intraoperative analgesia can increase postoperative complications, leading to poor prognosis and prolonged hospital stay [6]. In our previous study, the depth of analgesia of OFA in video-assisted thoracic surgery was continuously monitored using the pain threshold index (PTI) to determine whether non-opioid anesthesia could provide adequate analgesia [7]. The results showed that the OFA regimen could provide an equally sufficient intraoperative analgesia-nociception balance and lower depth of sedation with the wavelet index (WLI).

Controversies remain regarding the implementation of OFA, such as the optimal regimen, safety, and whether patients experience short and long-term benefits [6]. Beleoil et al. reported that bradycardia requiring atropine was more frequent in the dexmedetomidine group than in the remifentanil group. Three of the five cases of profound bradycardia in the dexmedetomidine group occurred during carbon dioxide insufflation during laparoscopic surgery. The level of intraoperative opioid-sparing could differ among surgical procedures, and not all analgesics may be safe in all procedures or patients. Following somatic, visceral and inflammatory components with pain originating from intestinal resection, abdominal insufflation and inflammation, laparoscopic radical colectomy (LRC) leads to peritoneal distention and damage and causes great perioperative pain. Opioids are widely used in LRC to provide effective analgesia and to inhibit sympathetic response. At present, few trials are investigating whether OFA, compared with traditional opioid anesthesia (OA), can provide a safe and effective analgesia-nociception balance with continuous PTI monitoring. We hypothesized that the use of OFA in patients receiving LRC could achieve the goals of analgesia, hypnosis, hemodynamic stability, and avoidance of opioid side effects.

In this prospective, randomized trial we tested the primary hypothesis that our OFA regimen, compared to traditional OA, could allow an efficacious intraoperative analgesia-antinociception balance with PTI monitoring. We further compared the difference between non-opioid and opioid anesthesia in the postoperative recovery of patients undergoing LRC.

## Methods

This was a randomized controlled and single-center clinical trial. The study protocol was approved by the Clinical Research Ethics Committee of the Hebei Medical University Fourth Hospital (#2,018,144; Chairperson Prof Guiying Wang) on Januray 20 2019. The study was registered a priori with the Chinese Clinical Trial Registry (www.chictr.org.cn, ChiCTR 1,900,021,223; on February 2 2019, principal investigator Xuelian Zhao). The eligible patient signed a written informed consent form after obtaining the consent of the participant.

### Participant eligibility

Inclusion criteria: Patients with an American Society of Anesthesiologists (ASA) physical status I or II, aged 18—65 years who were scheduled for elective LRC for colorectal cancer were recruited. The exclusion criteria were as follows: allergy to any experimental drug or its excipients; patients with central nervous system diseases (such as epilepsy, cerebral infarction, or cerebral hemorrhage history); a history of psychiatric illness; hepatic or renal impairment a history of chronic pain or alcohol or drug abuse; patients who were pregnant or breastfeeding women; patients who were unable to understand the pain assessment or use a patient-controlled analgesia (PCA) device; patients who were prescribed β-blockers and a heart rate (HR) < 50 bpm; and patients with body mass index (BMI) greater than 30 kg·m^−2^.

### Allocation and blinding

Patients were randomly allocated to the opioid-free anesthesia group (group OFA) or traditional opioid anesthesia group (group OA) using sealed opaque envelopes. The treatment arm was revealed on the morning of surgery. A computer-generated random allocation sequence was created by an independent investigator using Excel® version 2016 (Microsoft), with 1:1 allocation and random block sizes. Participants, surgical staff, and postoperative outcome assessors were blinded to group allocation, whereas anesthesia providers who did not participate in the assessment of the patients at any time could not be blinded to facilitate intraoperative anesthesia management.

### Electroencephalogram (EEG) measurement method

All patients underwent analgesia index (PTI) and sedation index (WLI) monitoring with a multifunction combination monitor HXD-I (Heilongjiang Huaxiang Technology Co., Ltd., Heilongjiang, China) after entering the operating room (see the report by An et al. [7]).

The PTI, a measure reflecting the antinociceptive state under general anesthesia, is based on EEG wavelet analysis ranging from 0 to 100. A PTI 40–60 is appropriate for intraoperative analgesia with higher values indicating a patient's lower pain tolerance. The WLI ranging from 0–100 shows the depth of sedation with changes in EEG signals. WLI < 35 over sedation; WLI 35–69 anesthesia, complete depression of consciousness; WLI 70–89 light narcosis/ sleep; WLI > 90 awake states.

### Ultrasound-guided bilateral paravertebral block (PVB)

Before the induction of GA, the patients in both groups underwent ultrasound-guided bilateral PVB in the prone position using an ultrasound machine (Voluson i, GE USA) and a high-frequency linear array probe. After the probe was placed at the level of the T10 -11 interspace, the apex of the paravertebral space was visualized. An 18G, 10 cm needle (Kangdelai, China) was inserted in the lateral-to-medial direction using an in-plane approach and advanced until the needle tip penetrated the internal intercostal membrane. The 15 ml mixed liquor (0.5% ropivacaine plus 0.2 μg·kg^−1^ dexmedetomidine in group OFA; 0.5% ropivacaine in group OA) was subsequently injected into the paravertebral space. The same procedure was repeated on the other side at T10—11. Sensory blockade was tested using the pinprick method. Patients were observed for adverse reactions associated with PVB over the next 15 min.

### Anesthetic management protocol

The OFA and OA regimens were based on our previously reported approaches (Table 1) [7].

**Table 1:** Time chart of anesthetic management

Group	Premedication	Pre-GA induction	GA Induction	GA Maintenance	GA Recovery	PCA (100 ml)
Group OFA	BPVB Ropivacaine (0.5%) Dexmedetomidine (0.2 μg/kg) 15 ml per side	Dex (0.6 μg/kg, 10 min) Dex (0.5 μg·kg^−1^·h^−1^, 30 min)	Dex (0.5 μg·kg^−1^·h^−1^) Ketorolac (30 mg) Propofol (2 mg/kg) Cisatracurium (0.2 mg/kg)	Dex ^a^ (0.5 μg·kg^−1^·h^−1^) Sevoflurane ^b^ (1%-3%) Cisatracurium (2–4 mg per 30 min)	Palonosetron 0.25 mg Neostigmine (up to 2 mg) Atropine (0.2–1 mg)	Dex (6 μg/kg) Ketorolac (180 mg)
Group OA	BPVB Ropivacaine (0.5%) 15 ml per side		Sufentanil (0.5 μg/kg) Propofol (2 mg/kg) Cisatracurium (0.2 mg/kg)	Remifentanil ^b^ (200–500 μg/h) Sevoflurane ^b^ (1%-3%) Cisatracurium (2 -4 mg per 30 min)	Namefene [8, 9] (0.05 mg) Palonosetron (0.25 mg) Neostigmine (up to 2 mg) Atropine (0.2–1 mg)	Dezocine (0.5 mg/kg) Ketorolac (180 mg)

*GA* General anesthesia, *BPVB* Bilateral paravertebral blockade, *Dex* Dexmedetomidine, *PCA* Patient control analgesia

^a^Infusion was stopped 60 min before end of surgery

^b^At end of surgery, inhalation and infusion were terminated

Following PVB, patients in the OFA group received an infusion of dexmedetomidine (0.6 μg·kg^−1^) and 0.5 mg atropine for 10 min, after which the infusion rate of dexmedetomidine was reduced to 0.5 μg·kg^−1^·h^−1^, terminating 60 min before the end of the surgery. All the patients received ketorolac (30 mg) for GA induction. Before the end of the operation, the patients received an intravenous bolus of 0.25 mg palonosetron to prevent post-operative nausea and vomiting (PONV), and the central vein was connected to a PCA machine (6 μg·kg^−1^ dexmedetomidine and 180 mg ketorolac added to 100 ml of saline at 2 ml·h^−1^ and the lock time was 15 min).

In the OA group, 0.5 μg·kg^−1^ sufentanil was administered intravenously (IV) for GA induction. To maintain GA, remifentanil was continuously infused at a rate of 4–10 μg·kg^−1^·h^−1^ and discontinued before skin closure. Before the end of the operation, the patient received 0.25 mg palonosetron IV, and PCA (0.5 mg·kg^−1^ dezocine and 180 mg ketorolac added to 100 ml with normal saline at 2 ml·h^−1^ and the lock time was 15 min).

Anesthesia induction was started 40 min after intravenous infusion of dexmedetomidine with 2 mg·kg^−1^ propofol and cis-atracurium 0.2 mg·kg^−1^ IV in all patients. Cis-atracurium (2- 4 mg every 30 min) and inhaled sevoflurane were administered to maintain anesthesia. Ventilation was controlled mechanically and adjusted to maintain the end-tidal CO_2_ value at 30—40 mmHg throughout surgery with 50% oxygen inhalation. The intraoperative carbon dioxide pneumoperitoneum pressure setting was maintained at 12 cmH_2_O. Air heating blankets were placed on any exposed parts of the body to maintain body temperature. After the operation, tracheal extubation was determined by the anesthesiologist when the patient reached a regular spontaneous breathing mode and WLI > 90. Postoperative rescue analgesic flurbiprofen axetil was injected intravenously based on the patient’s request.

### Outcomes

The primary outcome of this study was the intraoperative PTI readings recorded at after entering the operating room (T0, baseline value), before GA induction (T1), after intubation (T2), after incision (T3), 5 min after carbon dioxide pneumoperitoneum (T4), at the end of the operation (T5), and 5 min after extubation in the operation room (T6). Intraoperative secondary outcomes included WLI reading, mean arterial pressure (MAP), HR, pulse oxygen saturation (SpO_2_) at the same time points as when PTI was recorded, potential of hydrogen (pH), partial pressure of carbon dioxide, blood glucose concentration and lactic acid level from intraoperative blood gas after entering the operating room (G0, baseline value), before GA induction (G1), 1 h after surgery begun (G2), 2 h after surgery begun (G3), 3 h after surgery begun (G4), and 5 min after extubation in the operation room (G5). The total anesthetic consumption, extubation time, fluid infusion volume, and urine volume were measured and recorded. Postoperative secondary outcomes included VAS scores at 24 h, 48 h and 72 h after operation, time to flatus, PONV, length of stay and hospitalization cost. Intraoperative adverse events, including bradycardia, hypotension, and hypertension, and postoperative adverse events were recorded. Bradycardia was defined as a heart rate of < 45 bpm and was treated with intravenous atropine (0.5 mg). Hypotension (MAP < 60 mm Hg) was treated with norepinephrine. When the MAP was > 20% of the baseline value before induction, nitroglycerin was administered.

The potassium concentration between the groups was declared a secondary outcome in the trial registered on https://www.chictr.org.cn. However, we did not record intraoperative blood potassium values, because some patients had hypokalemia before the operation and received intravenous infusion of potassium chloride.

### Sample size calculation

The mean PTI at 5 min after pneumoperitoneum during the operation was 55.4 (7.3), as calculated in a previous pilot study of 15 patients with OA who underwent LRC. Therefore, we assumed that OFA could achieve an equal PTI. With a significance level of 0.05, a power of 0.85, upper equivalence limit of 5, lower equivalence limit of 15, true difference of 1 and standard deviation (SD) of 7, we calculated that each group should include 39 patients per group (PASS 11). Considering a loss- to- follow-up rate of approximately 20%, we needed to enroll 47 patients per group.

### Statistical analysis

All statistical analyses were performed using the chi-square independence test or Fisher's exact test to analyze categorical data. The unpaired Student's *t*- test was used to analyze the significance of the quantitative data. The Wilcoxon rank-sum test was used if the data were not normally distributed, as evaluated by the Shapiro–Wilk test. Descriptive parameters were expressed as mean values (SD). Highly skewed quantitative data are expressed as median interquartile range (IQR) with interquartile range.

The mean PTI readings at different time points were compared using an unpaired Student’s *t*-test and repeated-measures analysis of variance between the two groups. One-way analysis of variance was used to compare the mean PTI readings at each time point to the mean PTI at baseline within the OFA and OA groups using *Dunnett*’s multiple comparison test. Repeated measures analysis of variance, Student’s *t*-test, and one-way analysis of variance with Dunnett’s multiple comparison test were used to compare WLI, MAP, HR and SpO_2_ at seven-time points, and pH, the pressure of carbon dioxide (PaCO_2_), lactic acid levels, and blood glucose concentration at six-time points within and between groups. The mean VAS scores between the two groups were compared using the Mann–Whitney U test. Statistical significance was set at *P* < 0.05. All statistical analyses were performed using SPSS 17.0.

## Results

**Fig. 1:**
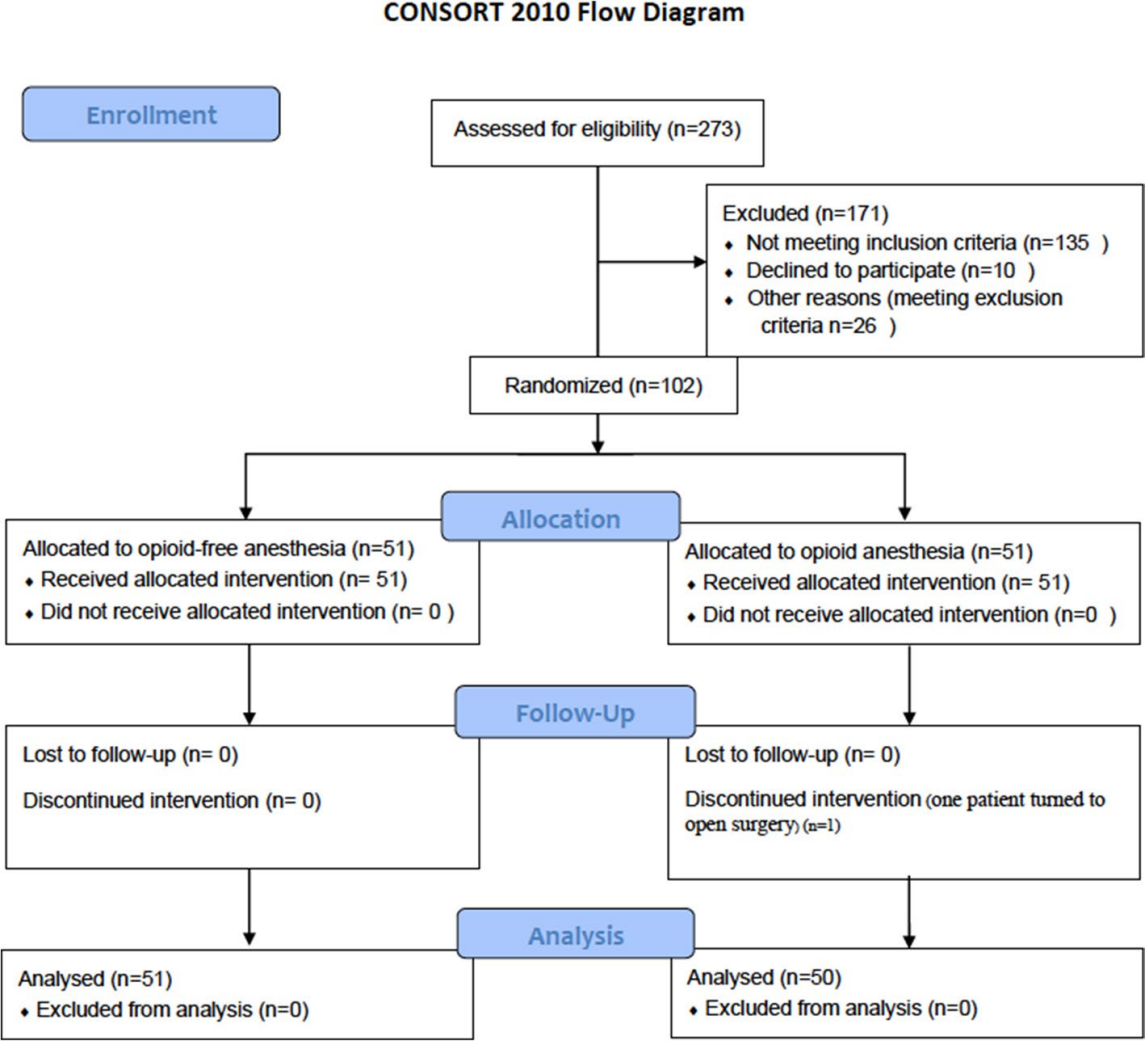
Study flow diagram. * Reasons for exclusion from the analysis: Group OA: 1-converted to open surgery

From February 2019 to November 2019, a total of 273 patients were included for eligibility assessment. In the end, 102 patients were selected for this clinical investigation. The two groups are comparable (Fig. 1). In the OA group, 1 case was converted to open operation, and 101 cases were suitable for analysis (Fig. 1). The groups were similar at baseline (Table 2).

**Table 2:** Patients characteristics and perioperative outcome variables

	Group OA *n* = 50	Group OFA *n* = 51	StD */ P*
Male (n, %)	22.0 (44.0)	28.0 (54.9)	0.109^¶^
Age (SD)	52.5 (10.2)	53.1 (8.6)	0.058^¶^
Body Mass Index (BMI, SD)	24.6 (2.9)	24.4 (3.1)	0.071^¶^
Duration of surgery (SD, min)	196.3 (51.5)	205.1 (48.4)	0.375^§^
Dexmedetomidine infusion time (SD, min)		154.5 (37.1)	-
Remifentanil infusion time (SD, min)	192.3 (51.8)		-
Fluid Infusion volumes (IQR, ml)	1900.0 (1500.0.0–2445)	2000.0 (1800.0–2245.0)	0.264*
Urinary volume (IQR, ml)	550.0 (400.0–750.0)	600.0 (360.0–800.0)	0.659*
Dexmedetomidine consumption (SD, ${\varvec{\mu}}$ g)	—	125.0 (33.0)	
Remifentanil consumption (SD, mg)	1.1 (0.4)	—	
Atropine consumption (IQR, mg)	0.9 (0.8–1.0)	1.0 (0.8–1.1)	0.028*
Norepinephrine consumption (IQR, ${\varvec{\mu}}$ g)	28.0 (0–160.0)	6.0 (0–65.0)	0.368*
Extubation time (SD, min)	10.9 (3.6)	15.0 (6.8)	< 0.001^§^
Urinary retention (n, %)	1 (2)	0	0.310^#^
Pruritus (n, %)	0	0	-
Postoperative nausea and vomiting (n, %)	2 (4)	0 (0)	0.149^#^
Bedtime (SD, h)	46.4 (11.5)	49.2 (11.1)	0.274^§^
Drinking time (SD, h)	46.2 (16.4)	44.4 (17)	0.593^§^
Time to passage of flatus (SD, h)	34.7 (18.6)	32.3 (19.9)	0.547^§^
Hospital discharge time (SD, day)	8.5 (2.9)	8.9 (2.8)	0.440^§^
Hospital expenses (SD, thousand Yuan)	80.4 (14.4)	78.6 (15.1)	0.535^§^

Data are presented as relative number of patients, mean ± standard deviation (SD), median (IQR)

^¶^ Standardized differences were calculated using Cohen d (JASP); ^*^ Wilcoxon rank-sum test; ^#^ Fisher’s exact test; ^§^ unpaired Student’s *t*-test

*SD* Standardized differences; Extubation time: time from the completion of intravenous neostigmine and/or nalmefene to the removal of the tracheal tube; Time to passage of flatus: the time from returning to the ward to the first breaking wind. Dexmedetomidine infusion time: the time from giving loading dose dexmedetomidine to stopping input. Remifentanil infusion time: the time from infusion remifentanil to stopping input

PTI readings, WLI readings, MAP, HR, SpO_2_, pH, PaCO_2_, lactic acid levels and blood glucose concentrations of two groups showed significant changes at different time points with single factor (time) repeated measurement analysis (*P* < 0.001). The results from multiple factors repeated measurement analysis of variance showed that PTI readings, MAP, SpO_2_, pH, PaCO_2_, lactic acid levels were not significantly different between the two anesthesia methods, while WLI readings, HR and blood glucose values were statistically significant between the two groups (*P* < 0.001).

**Fig. 2:**
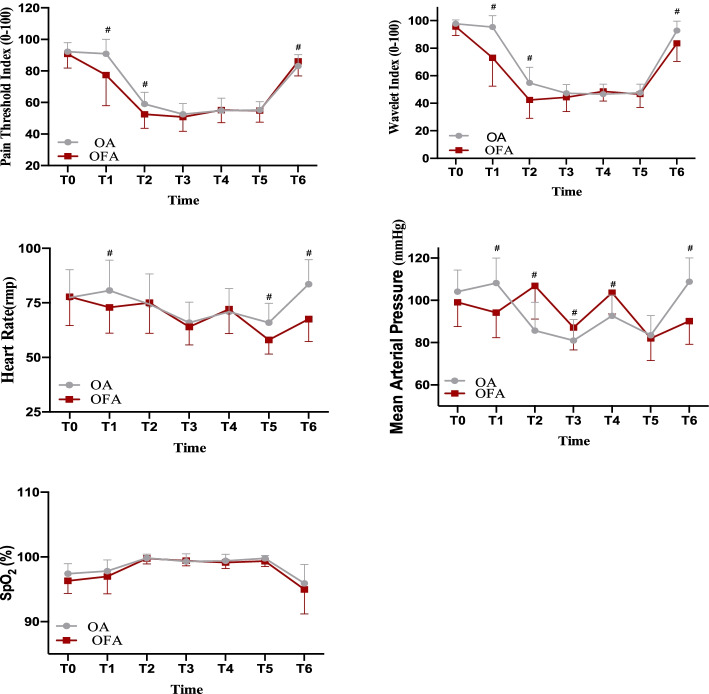
Changes in PTI, WLI, MAP, HR and SpO_2_. PTI: pain threshold index; BWI: brain wavelet index; MAP: mean arterial pressure; HR: heart rate; SpO_2_: pulse oxygen saturation. T0: baseline values, T1: before GA induction; T2: after intubation; T3: after incision; T4: 5 min after carbon dioxide pneumoperitoneum; T5: the end of operation; T6: 5 min after extubation in the operation room. *: unpaired Student’s *t*-test, *p* < 0.05 between groups

**Fig. 3:**
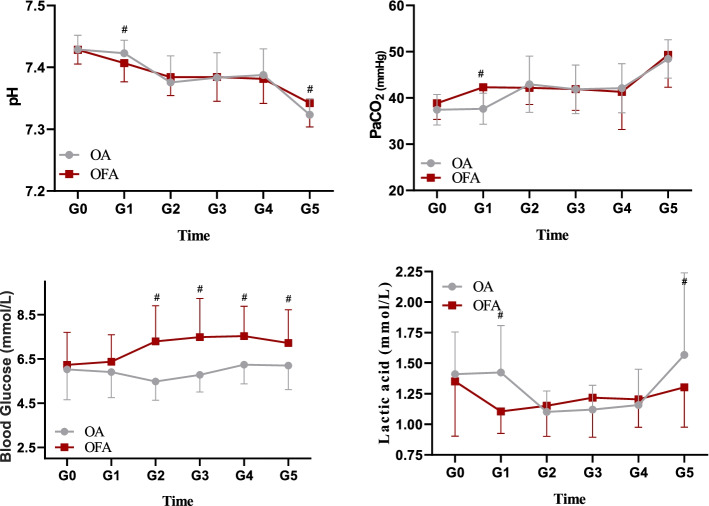
Changes in pH, partial pressure of carbon dioxide, blood glucose concentration and lactic acid level. G0: baseline values; G1: before GA induction; G2:1 h after surgery begun; G3:2 h after surgery begun; G4:3 h after surgery begun; G5: 5 min after extubation. PaCO_2_: arterial oxygen partial pressure. *: unpaired Student’s *t*-test, *p* < 0.05 between two groups

PTI readings and WLI readings in group OFA at T1 and T2 were significantly lower than those in group OA (*P* < 0.05, Fig. 2). The pH, lactic value at G1 in group OFA were lower than those in group OA (*P* < 0.05, Fig. 3). At G1, PaCO_2_ value in group OFA was significantly higher than that in group OA (*P* < 0.001, Fig. 3).

The visual analogue scale (VAS) scores at rest and on coughing between groups were not significantly different at 24 h, 48 h and 72 h after operation (*P* > 0.05, Table 3). The rescue analgesic consumption in the OFA group significantly decreased at 24 h and 72 h after operation (*P* < 0.05, Table 3).

**Table 3:** Comparison of VAS, number of PCA press and rescue analgesia consumption

Variable	Group	n	PO 24 h	PO 48 h	PO 72 h
VAS at rest (SD)	OA	50	1.7 (0.2)	1.8 (0.2)	1.8 (0.2)
	OFA	51	1.9 (0.2)	1.7 (0.1)	2.0 (0.2)
	*P*		0.36	0.59	0.36
VAS on coughing (SD)	OA	50	4.0 (0.3)	4.0 (0.3)	4.5 (0.2)
	OFA	51	4.5 (0.2)	4.3 (0.2)	4.9 (0.3)
	*P*		0.13	0.38	0.30
Number of PCA presses (IQR)	OA	50	0 (0–1)	0	0
	OFA	51	0 (0–2)	0	0
	*P*		0.671	-	-
Rescue analgesia consumption, (SD), mg	OA	50	140.0 (99.0)	138.0 (99.0)	144.0 (88.4)
	OFA	51	96.1 (91.6)	102.0 (96.9)	98.0 (95.0)
	*P*		0.021	0.066	0.021

*PO* Post operation, *VAS* Visual analogue scale, *OA* Opioid anesthesia, *OFA* Opioid-free anesthesia, *PCA* Patient control analgesia

Data are presented as relative number of patients, mean ± standard deviation (SD), median (IQR)

With unpaired Student’s t-test and Wilcoxon rank-sum test

The mean extubation time were significantly longer in group OFA (*P* < 0.001). There were not significantly different in urinary retention, pruritus, PONV, time to passage of flatus, hospital discharge time and hospital expenses between two groups. There was no local infection or hematoma at the puncture site 24 h after the operation. No patient died, was transferred to ICU and respiratory dysfunction, and no patient was intubated again after the tracheal intubation was removed. One case of atrial fibrillation (amiodarone treatment) and one case of hypotension (blood transfusion treatment) occurred in both the OFA group and the OA group. No patients in group OFA developed hypoxia (SpO_2_ < 90%) or bradycardia requiring treatment.

## Discussion

The results of this randomized, prospective study indicated that compared to opioid-based anesthesia, our OFA regimen with bilateral PVB in patients undergoing LRC could provide equally adequate analgesia and antinociception effects during operation, with better antinociception to intubation stimulation in patients (ASA I-II, 18–65 years old, BMI < 30 kg·m^−2^), guided by PTI monitoring. Non-opioid anesthesia can reduce the number of emergency analgesics at 24 h and 72 h after surgery, but there was no difference in VAS scores between the two groups after surgery. In addition, postoperative adverse reactions related to opioids in the OFA group, including PONV, urine retention, intestinal paralysis and pruritus, were not significantly different from those in the OA group.

The goal of providing OFA has been made possible by multimodal analgesia, based on the synergistic use of drugs with different modes of action, leading to additive pain management that works at different nociceptors along the pain pathway [10]. Continuous infusion of dexmedetomidine, inhalation of sevoflurane, intravenous bolus ketorolac and single-shot bilateral PVB (a mixture of bupivacaine and dexmedetomidine) were used to replaceOA in our study. PTI has emerged as a new method to monitor nociception and analgesia in unconscious patients [11–13]. Our results indicated that the PTI readings of group OFA were similar to those of group OA at the incision, 5 min after pneumoperitoneum, and at the end of the operation and were effectively maintained in the optimal range of 40 to 60, which is considered to predict adequate analgesia to nociceptive stimuli. Owing to its analgesic properties, dexmedetomidine has been used as an opioid substitute in various surgical procedures [10, 14–16]. Before anesthesia induction, a loading dose of dexmedetomidine (1 μg·kg^−1^) infusion showed a central anti-sympathetic effect that may spare the dose of anesthetics [17]. A meta-analysis revealed that dexmedetomidine combined with local anesthetics for the paravertebral block can significantly improve postoperative pain scores, prolong analgesia time, and reduce postoperative analgesic consumption [18]. In the present study, dexmedetomidine was intravenously infused and injected into the paravertebral space to exert analgesic effects through both central and peripheral mechanisms. Before induction of anesthesia, patients in the OFA group received 0.85 μg·kg^−1^ dexmedetomidine IV and 0.4 μg·kg^−1^ PVB over the period of 40 min, while the PTI readings decreased from 91 to 79 (*P* < 0.0001) and WLI readings reduced from 96 to 76 (*P* < 0.0001), which proved that dexmedetomidine has mild analgesic and moderate sedation properties. Beleoil et al. reported severe bradycardia in five patients associated with asystole, three of whom were in the non-opioid anesthesia group; four cases occurred during carbon dioxide insufflation for laparoscopic surgery [19]. The high incidence of severe bradycardia observed in this study is a consequence of the high dosage of dexmedetomidine of 1.2 μg·kg^−1^·h^−1^. The patients in our study received a 0.5 μg·kg^−1^·h^−1^ dexmedetomidine infusion, and no severe bradycardia occurred during the operation, even during carbon dioxide pneumoperitoneum. Ketorolac, a non-steroidal anti-inflammatory drug and an opioid-sparing analgesic was associated with a reduction in PCA opioid use, and was significantly better tolerated in terms of pruritus and nausea rates [20]. Shim and colleagues reported that intraoperative intravenous dexmedetomidine and ketorolac improved postoperative analgesia after robotic-assisted laparoscopic radical prostatectomy in patients who received rectus sheath block, and significantly decreased opioid requirement during the 24 h after surgery [21]. Wen and colleagues reported that compared with sufentanil-based analgesia for thoracoscopic surgery, dexmedetomidine combined with ketorolac in non-narcotic postoperative analgesia provided adequate and safe postoperative analgesia and reduced sufentanil consumption, analgesic-related complications, inflammation, and immunosuppression [22]. There was no difference in analgesic efficacy between bilateral PVB and epidural analgesia. Bilateral PVB has been successfully used in abdominal surgery without postoperative motor block or complications such as urinary retention [23]. Sun and colleagues reported that bilateral thoracic PVB combined with GA is associated with reduced rescue analgesia and morphine consumption compared to GA in off-pump coronary artery bypass grafting [24]. Sondekoppam and colleagues [25] reported that bilateral PVB provided non-inferior analgesia to thoracic epidural anesthesia in various abdominal surgeries requiring midline laparotomy. Systemically administered dexmedetomidine produces the same benefits as neuraxial dexmedetomidine in terms of prolongation and augmentation of spinal anesthesia without the potential for neurotoxicity and may therefore be preferable [26, 27]. Dose proportionality has been demonstrated within the therapeutic range for dexmedetomidine [28]. The reason fro the better antinociception to intubation response with lower PTI readings in the OFA group was that the patients received 1.25 μg·kg^−1^ for 40 min; prolonging the infusion time of dexmedetomidine before induction of anesthesia will help to exert its analgesic and sedative effects better.

In this study, patients in the OFA group who received non-opioid PCA regimen (dexmedetomidine and ketorolac) combined with bilateral PVB received less rescue analgesics than patients who received dezocine and ketorolac for PCA and PVB at 24 or72 h after surgery, while VAS scores showed no difference between groups. Tripathy and colleagues [3] reported that non-opioid nerve blocks (dexmedetomidine, ropivacaine and lignocaine) resulted lower VAS scores at 24 h after surgery and lesser morbidity for modified radical mastectomy with axillary clearance. Abd-Eishafy et al. reported that in patients who underwent elective video-assisted thoracic surgery, PVB with isobaric bupivacaine and dexmedetomidine (1 μg·kg^−1^) provided better pain relief during the early postoperative hours, and exerted a favorable effect on chronic postoperative pain [29]. There was no significant difference in the incidence of postoperative nausea and vomiting, urine retention, time to passage of flatus, or pruritus between the two groups.

Our study has several limitations. First, because the inhalation concentration of sevoflurane was adjusted many times during the operation, we could not accurately calculate the amount of sevoflurane that affected the intraoperative PTI and WLI. Second, this was a single-institution study with a small sample size, which may have resulted in selection bias. Third, patients in the OA group, were treated with dezocine, μ-receptor partial agonist antagonist, to manage postoperative pain and prevent remifentanil hyperalgesia. Fourth, intraoperative potassium levels as one of the secondary outcomes were not recorded because of intravenous potassium supplementation. Fifth, in the OFA group, a mixed solution of bupivacaine and dexmedetomidine was used for paravertebral block, which led to differences in the analgesic effect of the paravertebral block between the groups. Finally, further research is needed to evaluate the long-term survival benefits of avoiding opioids in patients with colorectal cancer.

## Conclusions

Our OFA regimen appears to be feasible for managing intraoperative nociception in LRC for colorectal cancer patients, with lower rescue analgesic consumption at 24 h and 72 h after operation. In addition, there was no significant difference in opioid adverse effects (PONV, urine retention, pruritus and intestinal paralysis) between non-opioid and opioid anesthesia.

## Data Availability

All data generated or analysed during this study are included in this published article [and its supplementary information files].

## Abbreviations

LRC: Laparoscopic radical colectomy
OA: Opioid based anesthesia
PONV: Postoperative nausea and vomiting
OFA: Opioid-free anesthesia
ICU: Intensive Care Unit
PTI: Pain threshold index
GA: General anesthesia
WLI: Wavelet index
VAS: Visual analogue scale
PVB: Paravertebral block
PCA: Patient-controlled analgesia
SD: Standard deviation
ASA: American Society of Anesthesiologists
IQR: Inter quartile range
BMI: Body mass index
